# Study on the relationship between vaginal dose and radiation-induced vaginal injury following cervical cancer radiotherapy, and model development

**DOI:** 10.3389/fpubh.2025.1585481

**Published:** 2025-05-19

**Authors:** Fei Kong, Ziqin Yan, Liying Gao, Jianping Long, Xu Wang, Zhangcai Zheng

**Affiliations:** ^1^Gansu Provincial Maternal and Child Health Care Hospital (Gansu Provincial Central Hospital), Lanzhou, China; ^2^Cloud Gansu Technology Co., Ltd., Lanzhou, China

**Keywords:** cervical cancer, radiotherapy, vaginal dose, radiation-induced vaginal injury, deep learning

## Abstract

**Objective:**

This study investigates the relationship between vaginal radiation dose and radiation-induced vaginal injury in cervical cancer patients, with the aim of developing a risk prediction model to support personalized treatment strategies.

**Methods:**

A retrospective analysis was performed on the clinical data of 66 cervical cancer patients treated between December 2022 and December 2023. The Synthetic Minority Over-sampling Technique (SMOTE) was employed for data augmentation. Univariate and multivariate analyses were conducted to identify key factors influencing radiation-induced vaginal injury, and five distinct algorithms were applied to develop predictive models. The AUC/ROC metric was used to assess the performance of the models.

**Results:**

Univariate analysis revealed significant associations between the posterior-inferior border of the symphysis (PIBS) point dose and brachytherapy dose with radiation-induced vaginal injury (*p* < 0.05). Multivariate analysis confirmed PIBS point dose, brachytherapy dose, age, external beam radiation dose, and vaginal involvement as significant factors. A neural network algorithm was chosen to construct the radiation-induced vaginal injury model, which was subsequently developed into an online tool.

**Conclusion:**

The developed predictive model can assess the risk of radiation-induced vaginal injury, thereby facilitating the development of individualized radiotherapy plans.

## 1 Introduction

Cervical cancer is among the most prevalent malignancies affecting women worldwide, with high incidence and mortality rates compared to other gynecological cancers (1). According to the World Health Organization (WHO), in 2020, approximately 600,000 new cases and 347,000 deaths occurred globally due to cervical cancer (2). In China, the incidence of cervical cancer is also rising, presenting a significant threat to women’s health (3). Radiotherapy, a primary treatment modality for cervical cancer, plays a critical role in improving patients’ survival rates and quality of life.

In recent years, advancements in radiotherapy techniques, such as volumetric modulated arc therapy (VMAT) and image-guided radiotherapy (IGRT), have significantly improved local control and survival rates for cervical cancer patients (4). However, radiotherapy inevitably causes damage to surrounding normal tissues (5). Radiation-induced vaginal injury is a common complication of radiotherapy for cervical cancer, severely affecting patients’ quality of life (6, 7). Despite these advancements, the relationship between vaginal dose and radiation-induced injury remains unclear, and there is a lack of quantitative models capable of accurately assessing the risk of vaginal injury (8, 9). This uncertainty complicates the process of individualizing radiotherapy plans in clinical practice, making it challenging to minimize the occurrence of vaginal injury. Several studies have attempted to evaluate the correlation between vaginal dose and radiation-induced morbidity through dosimetric analysis, but the results have been somewhat limited (5, 10, 11). Moreover, strategies for the prevention and management of radiation-induced vaginal complications still require further optimization (12, 13).

Consequently, investigating the relationship between vaginal dose post-radiotherapy and radiation-induced vaginal injury holds significant clinical relevance. Developing a precise predictive model for vaginal injury risk facilitates the creation of individualized treatment plans, supporting the radiotherapy team in accurately controlling vaginal dose during treatment planning. This approach aims to minimize vaginal damage and enhance patient quality of life. Furthermore, this research will provide a theoretical foundation for future treatment and prevention strategies, thereby contributing to advancing radiotherapy techniques for cervical cancer.

## 2 Materials and methods

### 2.1 General information

Patients with cervical cancer who underwent brachytherapy at our institution between December 2022 and December 2023 were retrospectively selected. The inclusion criteria were as follows: (1) a confirmed diagnosis via pathological examination; (2) availability of complete clinical and follow-up data; and (3) receipt of brachytherapy treatment. The exclusion criteria included: (1) severe dysfunction of major organs (heart, lungs, or kidneys); (2) presence of other concurrent malignancies; (3) severe bone marrow suppression; (4) estimated survival time of less than 3 months; (5) distant metastasis (excluding stages IVa and IVb); and (6) pregnancy or lactation.

A total of 66 cervical cancer patients were enrolled in this study, including 35 patients who underwent definitive radiotherapy and 31 patients who had received postoperative treatment. The median age of the cohort was 55 years (28–79 years). Histopathological subtypes included squamous cell carcinoma (*n* = 60), adenocarcinoma (*n* = 4), and adenosquamous carcinoma (*n* = 2). According to the 2018 FIGO staging system, the distribution was as follows: stage I (*n* = 8), stage II (*n* = 29), and stage III (*n* = 29). Vaginal involvement was assessed as follows: no involvement (*n* = 3), upper one-third involvement (*n* = 48), middle one-third involvement (*n* = 13), and lower one-third involvement (*n* = 2). This study was approved by the Institutional Review Board of our hospital (Approval Number: (2024) GSFY No. [62]), All patients provided written informed consent prior to participation in the study.

### 2.2 Methods

#### 2.2.1 Radiotherapy

A cohort of 66 patients diagnosed with cervical cancer underwent a combined external beam radiation therapy (EBRT) and intracavitary brachytherapy regimen at our institution. EBRT was delivered using volumetric modulated arc therapy (VMAT) with the Precise Treatment System from Elekta, Sweden. Target delineation and treatment planning adhered to the guidelines outlined in the International Commission on Radiation Units and Measurement (ICRU)-50 report (14). The total EBRT dose ranged from 45 to 50.4 Gy, delivered in 25 to 28 fractions, five times per week. Following EBRT, intracavitary brachytherapy was administered using the micro-selectron-HDR 192Ir afterloading system from Nucletron, Netherlands. For patients undergoing definitive radiotherapy, the total brachytherapy dose ranged from 24.0 to 30.0 Gy, delivered in 4 to 5 fractions, twice per week. The cumulative total dose of definitive radiotherapy was converted to an equivalent dose in 2 Gy fractions (EQD2) for calculation and dose assessment, with a final A-point total dose ranging from 77.0 to 90.4 Gy. Postoperative patients received a total brachytherapy dose of 11 Gy, delivered in 2 fractions, once per week. The cumulative total dose of postoperative radiotherapy was converted to EQD2 for calculation and dose assessment, resulting in a final A-point total dose ranging from 59.21 to 64.61 Gy. The entire radiotherapy course was completed within 8 weeks for all patients.

#### 2.2.2 Diagnostic criteria for radiation-induced vaginal injury

The diagnostic criteria for radiation-induced vaginal injury are based on the National Cancer Institute (NCI) Common Terminology Criteria for Adverse Events (CTCAE) version 4.0 (15). These criteria are classified into four grades based on objective assessment:

- Grade I: Vaginal length >2/3 of normal length, no symptoms of dryness or bleeding, superficial ulcers or necrosis ≤1 cm^3^, and mild vaginal atrophy.
- Grade II: Vaginal length 1/3 to 2/3 of normal length, contact bleeding present, no dryness, superficial ulcers or necrosis >1 cm^3^, and more severe vaginal atrophy.
- Grade III: Vaginal length <1/3 of normal length, dryness, intermittent bleeding, deep ulcers, and severe vaginal atrophy.
- Grade IV: Vaginal closure, fistula formation, diffuse atrophy, often accompanied by bleeding.

#### 2.2.3 Follow-up visits

After completing all treatments, patients were regularly followed up in the outpatient clinic. The follow-up intervals were as follows: the first review took place 1 month after the completion of treatment during the first year, followed by reviews every 3 months thereafter. In the second year, follow-ups occurred every 6 months. The follow-up included gynecological examinations, as well as necessary blood and imaging tests, and the assessment and recording of vaginal injury based on both subjective and objective criteria. Patients with a shorter time to completion of treatment may have undergone additional follow-up visits during the study period, based on the data collected during the last examination at the first visit.

#### 2.2.4 PIBS point dose

**Figure 1:**
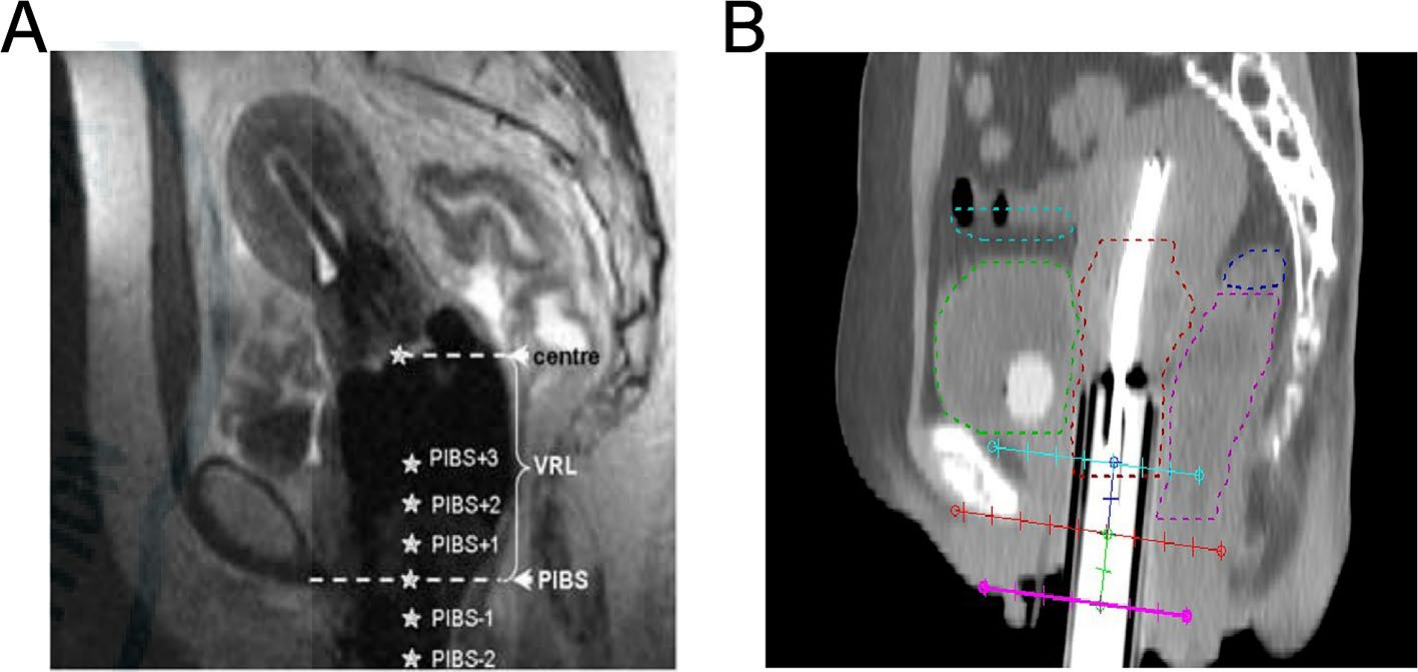
PIBS point dose: **(A)** shows the location of PIBS in cervical cancer; **(B)** shows the location of the selected PIBS point dose in this study.

PIBS system is described in the literature (16). It is the point where the PIBS line intersects perpendicularly with the uterine applicator, representing the middle and lower thirds of the vaginal junction, as well as the level of the clitoris and anal sphincter, as shown in Figure 1A. The PIBS-2 point refers to the location of the 2 cm lateral to the pedicle along the uterine canal, marking the vaginal opening. The PIBS+2 point refers to the location of the 2 cm cephalad along the uterine canal, representing the midpoint of the vagina. Since the mucosal surface dose is prone to overlap with the target area, potentially leading to overdose, the submucosal 5 mm depth was used as the dosimetry point in this study, as shown in Figure 1B.

#### 2.2.5 Data augmentation

In the field of machine learning and data analytics, data augmentation is a crucial technique used to expand datasets through various methods, thereby improving the generalization ability and robustness of the model. The data augmentation process primarily employs the Synthetic Minority Over-sampling Technique (SMOTE), a widely used synthetic sample generation method designed to address imbalanced datasets. The core principle of SMOTE is to increase the number of minority class samples by generating synthetic samples, thus balancing the distribution of classes in the dataset (17). The advantage of this method lies in its ability to significantly increase the amount of data without altering the original characteristics of the dataset, thus providing richer information for model training (18). The algorithmic steps of SMOTE are strictly adhered to throughout the process to ensure that the generated synthetic samples are both representative and maintain the distributional characteristics of the original data. This method not only increases the size of the dataset but also preserves the original characteristics of the data, providing a more reliable foundation for subsequent model construction and validation.

#### 2.2.6 Data preparation and enhancement

**Figure 2:**
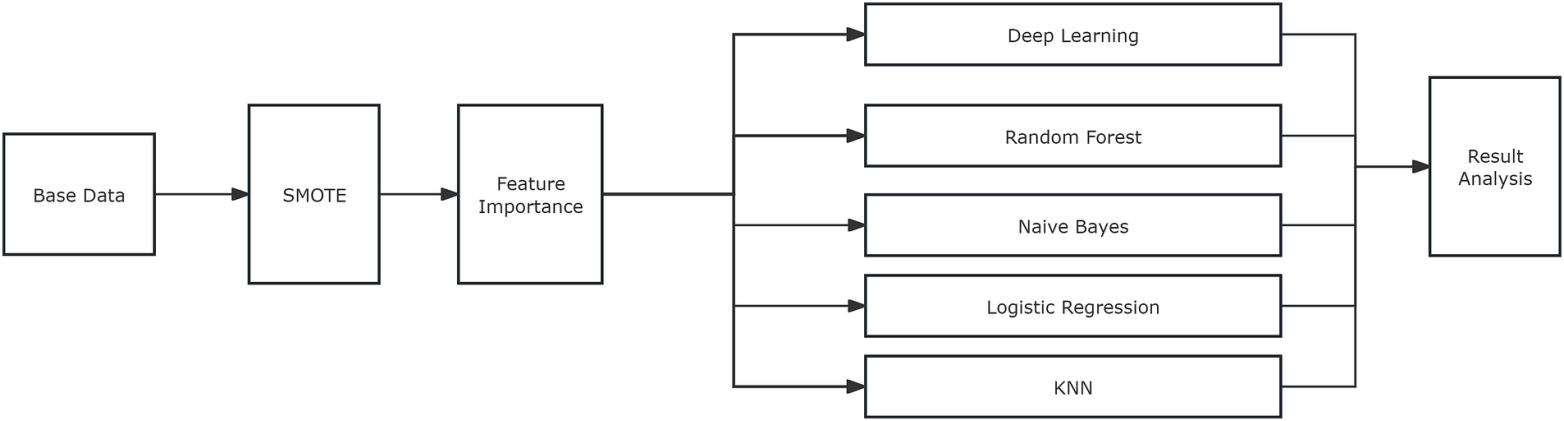
Flowchart for model construction.

In this study, clinical data from 66 cervical cancer patients were collected, including information on patients’ age, pathological type, clinical stage, vaginal involvement, surgical history, external irradiation dose, PIBS point dose, PIBS point +2 cm dose, and PIBS point −2 cm dose. To increase the dataset size and improve the model’s generalization ability, the SMOTE method was employed to augment the data, expanding the number of cases from 66 to 20,000. The specific process is illustrated in Figure 2.

##### 2.2.6.1 Feature selection and dimensionality reduction

After data augmentation, the Feature Importance algorithm was employed to assess the impact of each feature on vaginal radiation damage (19). The Feature Importance algorithm assesses how much input features impact model predictions. It uses specific calculation or evaluation methods to quantify each feature’s importance, aiding in model decision - making understanding and feature selection. Based on the algorithm’s results, we rank them. Based on the results of the algorithm, features with minimal influence on model predictions were removed, including “pathology” (type of pathology), “surgery” (whether surgery was performed), “PIBS+2 cm” (PIBS point +2 cm dose), “PIBS-2 cm” (PIBS point −2 cm dose), and “clinical stages.” This process helps to simplify the model and enhance its predictive performance.

##### 2.2.6.2 Model construction and training

Five deep learning and machine learning algorithms were used to construct the prediction model, including Deep Learning (20), Random Forest (21), Naïve Bayes (22), Logistic Regression (23), and K-nearest Neighbor (KNN) (24).

###### 2.2.6.2.1 Deep learning

This is a two-layer feed-forward neural network consisting mainly of an input layer, a hidden layer, and an output layer. The input data first passes through a fully connected layer (linear layer) and is mapped to the feature space of the hidden layer. Then, a nonlinear transformation is introduced through the ReLU activation function (25), and the prediction results are output through another fully connected layer.

###### 2.2.6.2.2 Random Forest model

This is an ensemble learning method used for classification or regression tasks by constructing multiple decision trees and combining their results through voting or averaging. Each decision tree is trained using a randomly sampled subset of the data and a subset of features, which increases the diversity of the model, reduces the risk of overfitting, and provides high accuracy and robustness.

###### 2.2.6.2.3 Naïve Bayes

This is a classification algorithm based on Bayes’ theorem, which assumes that features are independent of each other (i.e., the “naïve” assumption). It calculates the conditional probability for each category and selects the category with the highest probability as the prediction.

###### 2.2.6.2.4 K-nearest neighbor (KNN)

This is a non-parametric, example-based learning method that calculates the distances between the sample to be predicted and all samples in the training set. It then identifies the k closest samples (i.e., the “neighbors”) and classifies or averages the categories or values of these neighbors (classification for categorical variables, regression for numerical values). KNN is simple and intuitive but has high computational complexity and is sensitive to data distribution and noise.

###### 2.2.6.2.5 Logistic regression

This is a linear model for binary classification tasks that represent the probability of a sample belonging to a category by mapping the output of a linear function to the interval [0, 1] using a sigmoid activation function. Logistic regression is highly interpretable, with parameter learning based on maximum likelihood estimation.

#### 2.2.7 Model optimization and validation

In this study, the area under the curve (AUC) was used to evaluate the performance of the risk prediction model. AUC refers to the area under the receiver operating characteristic (ROC) curve, which demonstrates the sensitivity and specificity of the model’s predictions. The AUC value allows for a quantitative comparison of the model’s prediction performance (26). A higher AUC value indicates better predictive performance.

#### 2.2.8 Statistical methods

Statistical analysis was performed using Python 3.9.12. Categorical data were expressed as rates, and the *χ*^2^ test was used for comparisons.

## 3 Results

### 3.1 Single-factor analysis of factors affecting the risk of vaginal radiation injury due to radiotherapy in cervical cancer patients

Among the 66 cervical cancer patients treated with brachytherapy, 61 patients experienced vaginal radiation injury, resulting in an overall incidence rate of 92.4% (61/66). The distribution of vaginal radiation injury severity was as follows: grade I, 56% (37/66); grade II, 27.3% (18/66); and grade III, 9% (6/66). The difference between these grades was statistically significant (*χ*^2^ = 123.57, *p* < 0.05); no cases of grade IV vaginal radiation injury were observed. A single-factor analysis using the χ^2^ test, after combining patients with grade I, II, and III vaginal radiation injury, revealed that the PIBS point dose (*χ*^2^ = 15.359, *p* = 0.002) and brachytherapy dose (*χ*^2^ = 22.536, *p* = 0.007) significantly affected the occurrence of vaginal radiation injury. In contrast, no significant associations were found between the occurrence of vaginal radiation injury and factors such as patient age, pathology type, clinical stage, vaginal involvement, surgical history, external irradiation dose, PIBS+2 cm dose, and PIBS-2 cm dose (all *p* > 0.05) (see Table 1).

**Table 1:** Results of single-factor analysis affecting the occurrence of vaginal radiation damage after radiotherapy in cervical cancer patients.

Clinicopathologic feature	Total case	Vaginal radiation injury due to radiotherapy			Total		*χ* ^2^	*p-*value
		Grade I	Grade II	Grade III	Case	Percent %		
Age(years)							91.288	0.827
≤ 55	37	25	6	3	34	91.9		
> 55	29	12	12	3	27	93.1		
Pathology type							7.074	0.314
Squamous cell carcinoma	60	34	16	6	56	93.3		
Adenocarcinoma	4	2	2	0	4	100		
Adenosquamous carcinoma	2	1	0	0	1	50.0		
Clinical stage							7.799	0.253
Stage I	8	6	0	0	6	75.0		
Stage II	29	15	9	3	27	93.1		
Stage III	29	16	9	3	28	96.6		
Vaginal involvement							54.443	0.688
Uninjured	3	0	1	0	1	33.3		
Upper one-third	48	29	13	3	45	93.6		
Middle one-third	13	7	4	2	13	100		
Lower one-third	2	1	0	1	1	100		
Operation							47.965	0.678
Radical radiation therapy	35	14	14	6	34	97.1		
Postoperation	31	23	4	9	27	87.1		
External irradiation dose							6.126	0.409
45Gy	31	16	7	4	27	87.1		
50Gy	10	8	2	0	10	100		
50.4Gy	25	13	9	2	24	96.0		
Brachytherapy dose^a^							22.536	0.007
≤ 24Gy	32	24	4	0	28	87.5		
> 24Gy	34	12	14	6	32	94.1		
PIBS point dose							15.359	0.002
≤ 5Gy	31	23	3	0	26	83.9		
> 5 Gy	35	13	15	6	34	97.1		
PIBS+2 cm dose							61.443	0.628
≤ 10Gy	34	24	4	1	29	85.3		
> 10 Gy	32	13	14	5	32	100		
PIBS-2 cm dose							54.110	0.659
≤ 3Gy	38	27	5	1	33	86.9		
> 3Gy	28	10	13	5	28	100		

^a^ is the dose at the point A.

### 3.2 Multifactorial analysis of factors affecting the risk of vaginal radiation injury due to radiotherapy in cervical cancer patients

**Figure 3:**
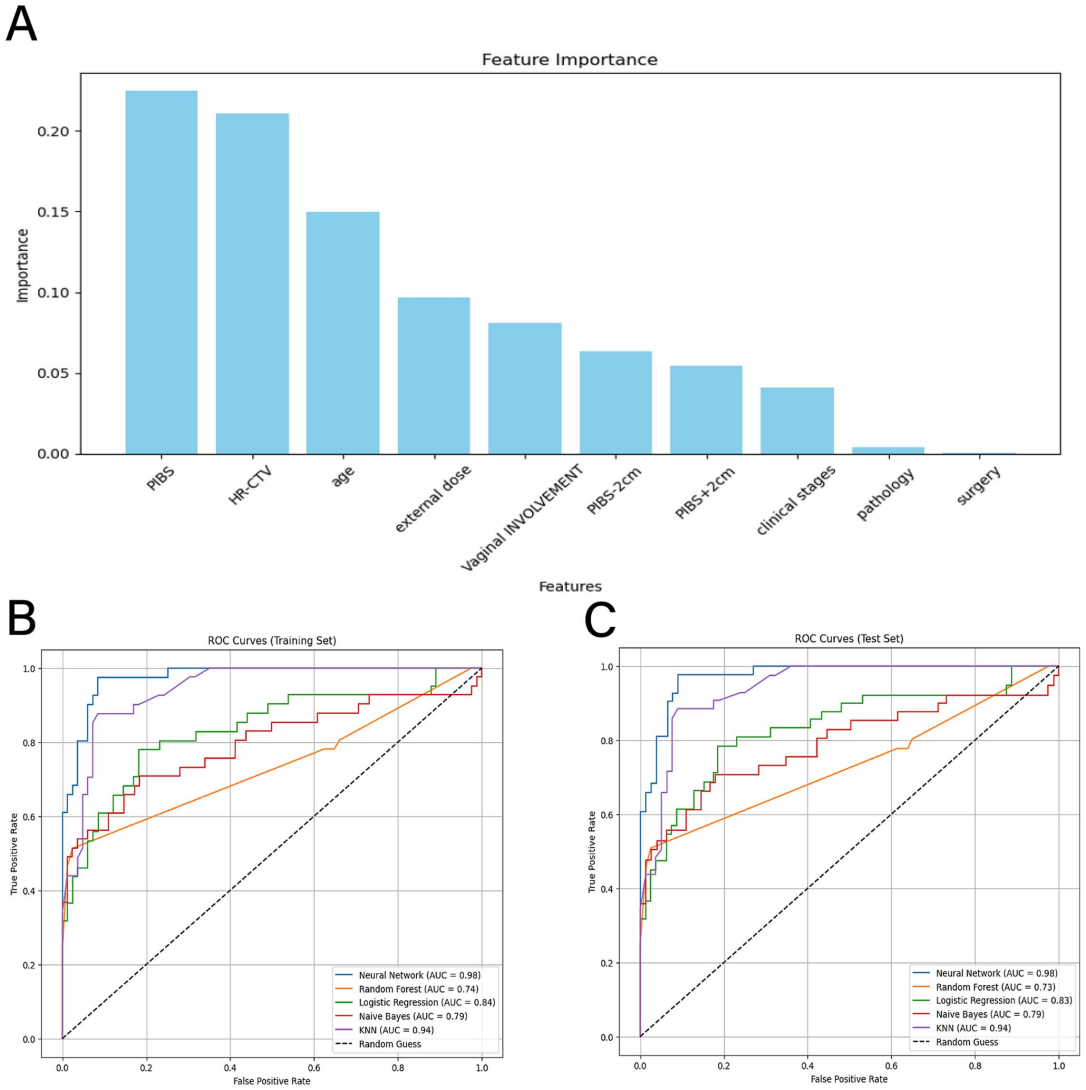
**(A)** graph shows the percentage of Feature Importance; **(B)** (training set) and **(C)** (validation set) show the ROCAUC evaluation model, with the black dashed center as the reference line, and the neural network algorithm model in blue, the random forest algorithm model in orange, the logistic regression algorithm model in green, the plain Bayesian algorithm model in red, and the KNN algorithm model in purple.

In this study, to increase the dataset size and improve the generalization ability of the model, the SMOTE method was used to augment the data, expanding the number of cases from 66 to 20,000. The SMOTE method balances the distribution of categories in the dataset by generating synthetic samples, thereby increasing the number of samples from underrepresented categories. The patient’s age, pathological type, clinical stage, vaginal involvement, surgical history, external irradiation dose, PIBS point dose, PIBS point +2 cm dose, PIBS point -2 cm dose, and other factors were used as independent variables, with the degree of vaginal radiation injury serving as the dependent variable. The Feature Importance algorithm was applied to identify the factors affecting vaginal radiation injury due to radiotherapy in cervical cancer patients. This algorithm provided the importance ratios for all the factors, and the variable assignments are shown in Table 2. The analysis results indicate that the patient’s PIBS point dose, brachytherapy dose, age, external irradiation dose, and vaginal involvement accounted for a relatively large proportion of the vaginal radiation injuries due to radiotherapy in cervical cancer patients, and these were identified as key factors in radiologic vaginal injury (see Figure 3A).

**Table 2:** Assignment of variables.

	Variables	Assignment
Dependent variable	Degree of vaginal radiation injury	0 = Uninjured, 1 = Grade I, 2 = Grade II, 3 = Grade III
Independent variables	Age	Actual value
	Pathology type	0 = squamous cell carcinoma, 1 = adenocarcinoma, 2 = adenosquamous carcinoma
	Clinical stage	0 = Stage I, 1 = Stage II, 2 = Stage III
	Vaginal involvement	0 = Uninjured, 1 = Upper one-third, 2 = Middle one-third, 3 = Lower one-third
	Operation	0 = Radical radiation therapy, 1 = Postoperation
	External irradiation dose	Actual value
	Brachytherapy dose	
	PIBS point dose	
	PIBS+2 cm dose	
	PIBS-2 cm dose	

### 3.3 Efficacy analysis of the model for radiation-induced vaginal injury

The five factors identified as significantly associated with radiation-induced vaginal injury were used to construct the model for radiation-induced vaginal injury. In this study, 80% of the augmented dataset was used as the training set, while 20% was used as the test set. Five learning algorithms—Random Forest, Naïve Bayes classification, k-nearest neighbor (KNN), Neural Network, and logistic regression—were applied for modeling using the optimal subset of features, with the AUC/ROC metric used to evaluate model efficacy. As shown in Table 3 and Figures 3B,C, the neural network model achieved the highest AUC values of 0.9765 for the training set and 0.9751 for the test set, indicating the model’s superior performance in predicting vaginal radiation injury following radiotherapy for cervical cancer. The k-nearest neighbor model followed closely, with AUC values of 0.9433 for the training set and 0.9422 for the test set. The logistic regression model also demonstrated high AUC values of 0.8353 for the training set and 0.8348 for the test set. The Random Forest and Naïve Bayes models had relatively lower AUC values but still demonstrated some predictive power, with AUC values of 0.7359 and 0.7349 for the training and test sets (Random Forest), and 0.7948 and 0.7903 for Naïve Bayes, respectively. Therefore, the neural network model was ultimately selected to construct the prediction model for vaginal radiation injury following radiotherapy for cervical cancer.

**Table 3:** AUC values for the training and test sets.

Model	Training sets AUC	Test sets AUC
Neural Network	0.9765	0.9751
Random Forest	0.7359	0.7349
Logistic Regression	0.8353	0.8348
Naïve Bayes classification	0.7948	0.7903
k-nearest neighbor	0.9433	0.9422

### 3.4 Construction of an online program for predicting the risk of vaginal radiation injury

**Figure 4:**
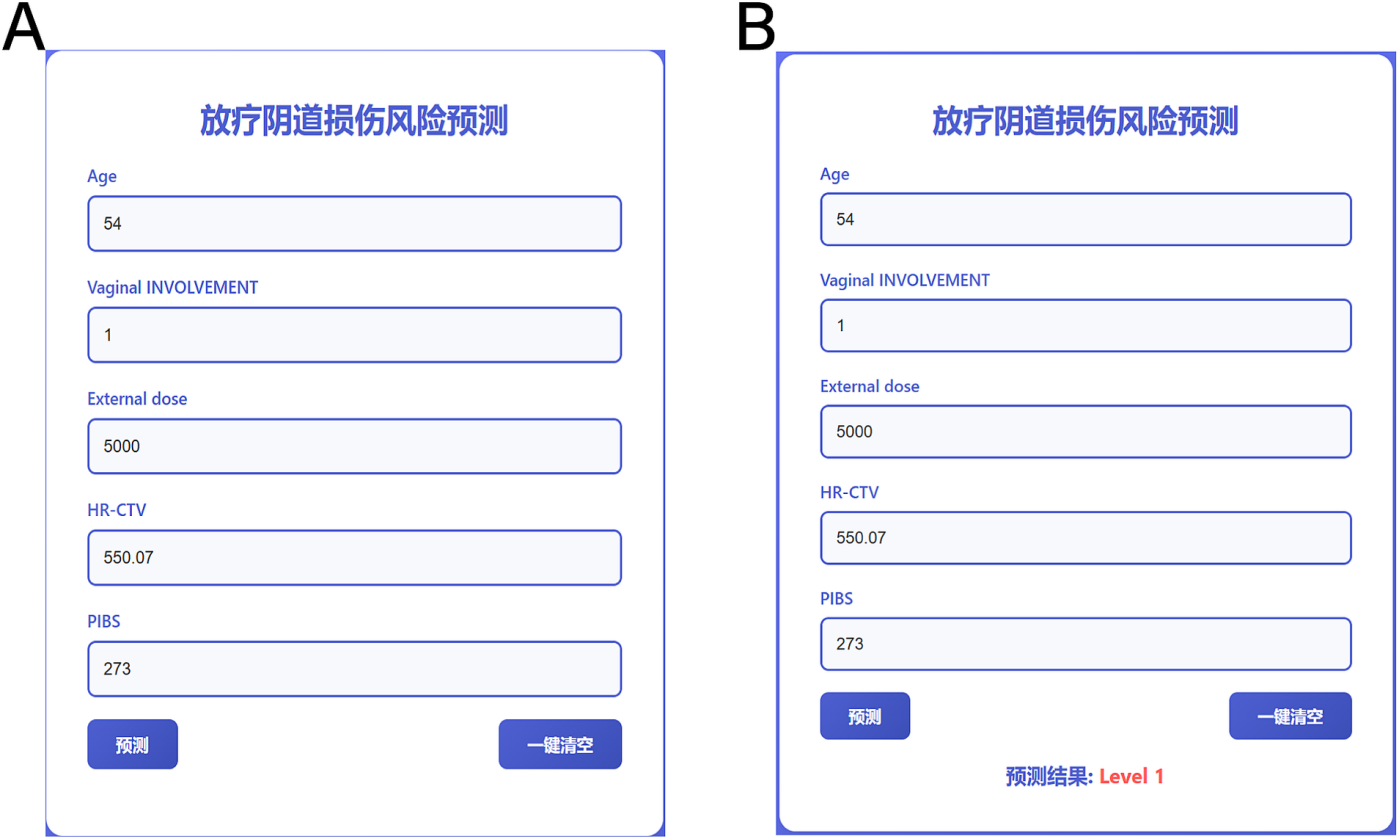
Online program for predicting the risk of radiologic vaginal injury. **(A)** is the input data and **(B)** is the output result.

To make the model more intuitive and clinically applicable, we further developed it into an online program. An online program serves as a visualization tool that integrates multiple predictors, allowing for a simple numerical representation of the contribution of each factor to the prediction result. In this study, we created the corresponding online program based on the model’s output. Users only need to input the values for PIBS point dose, brachytherapy dose, age, external irradiation dose, and vaginal involvement to obtain the probability of a patient’s risk of developing vaginal radiation injury, as shown in Figure 4.

## 4 Discussion

Vaginal radiation injury is a common complication following radiotherapy in female patients with pelvic and abdominal tumors. Symptoms such as vaginal dryness, bleeding, pain, vaginal stenosis, ulceration, mucosal atrophy, and pain during sexual intercourse due to vaginal atresia can significantly affect patients’ self-image, particularly in younger patients. This can damage their self-esteem and result in a substantial decline in the quality of life for those who have survived cervical cancer. During radiotherapy for cervical cancer, the vagina is both a target organ for treatment and a critical organ at risk. When subjected to high radiation doses, radiation damage is almost inevitable (27).

In recent years, researchers have focused more on radiation proctitis and cystitis, with numerous studies exploring pathogenic factors, clinical manifestations, and prophylactic treatments. However, little attention has been given to radiation-induced vaginal injuries (28, 29). The PIBS point reference system, along with PIBS ± 2 cm and vaginal reference length (VRL), for assessing vaginal radiation doses may offer a more reasonable approach, as proposed by the latest study conducted by the International Study Group on MRI-guided Brachytherapy for Locally Advanced Cervical Cancer in Europe (16). Westerveld et al. (16) conducted a series of studies using the PIBS system, examining MRI-guided internal irradiation treatment in 59 patients with cervical cancer. They found that 20% of the patients experienced vaginal damage, with PIBS, PIBS +2 cm, and PIBS -2 cm doses of 6.4, 32.9, and 2.2 Gy, respectively (*p* < 0.05), with the PIBS +2 cm dose showing the most significant difference. Dankulchai et al. (11) retrospectively examined the correlation between vaginal dose-volume toxicity and the 11 o’clock position of the vagina in cervical cancer radiotherapy. Their analysis revealed statistically significant differences in PIBS + 2, PIBS, and PIBS-2 doses across grades 1 to 3 of vaginal stenosis, with notable probability coefficients. Furthermore, significant associations were identified between factors such as age, tumor size, the involvement of the lower third of the vagina, and the incidence of vaginal toxicity. Kirchheiner et al. (27) conducted a study on 630 patients with locally advanced cervical cancer, identifying external beam radiation dose (HR = 1.770, *p* = 0.056) and vaginal involvement (HR = 2.259, *p* < 0.001) as risk factors for vaginal stenosis. These findings suggest that PIBS point dose, age, external beam radiation dose, and vaginal involvement are crucial risk factors for vaginal injury following cervical cancer radiotherapy, a conclusion consistent with the present study. In contrast, Wang et al. (30) performed a prospective study involving 351 Chinese patients with cervical cancer, focusing on the relationship between vaginal dose and vaginal stenosis (VS). In their univariate Cox regression analysis, PIBS point dose and PIBS + 2 cm dose were identified as risk factors for VS. However, in their multivariate Cox regression analysis, these doses were not significant predictors of VS, and no statistically significant differences were observed between PIBS point dose, PIBS ± 2 cm dose, and vaginal stenosis. Instead, VRL was confirmed as a significant risk factor for VS, a finding that contrasts with the results of this study. This discrepancy may be attributable to the strong correlation between PIBS + 2 cm dose, PIBS point dose, and VRL, which was not further explored in the current study. Additionally, Wang et al.’s (30) research suggested that VRL in Chinese patients is relatively shorter than in Western populations, and Chinese patients with cervical cancer receive higher total doses of external beam radiation therapy (EBRT) and brachytherapy (BT) in the upper two-thirds of the vagina. Consequently, evaluating vaginal injury using PIBS point dose remains a reasonable approach within this context.

This study is innovative in several aspects regarding the relationship between vaginal dose and radiation-induced vaginal injury after radiotherapy for cervical cancer. First, the original data from 66 cases were augmented to 20,000 cases using a data enhancement technique, significantly increasing the dataset size and improving the model’s generalization ability and robustness. This process utilized the SMOTE method, which effectively balanced the distribution of categories in the dataset and helped prevent overfitting due to the initially limited data volume. Second, this study comprehensively employed a variety of deep learning and machine learning algorithms, including deep learning, Random Forest, Naïve Bayes, logistic regression, and k-nearest neighbor, to construct and compare risk prediction models for vaginal radiation injury, ultimately selecting the neural network model, which demonstrated the best performance. Furthermore, this study not only identified key factors such as PIBS point dose, brachytherapy dose, age, external irradiation dose, and vaginal involvement but also transformed the complex model results into an intuitive, visualization tool in the form of an online program. This tool enables clinicians to quickly and accurately assess the risk of vaginal radiation injury in patients, providing strong support for the development of individualized radiotherapy plans. In clinical practice, the prevention and management of vaginal injury during radiotherapy for cervical cancer remain critical challenges. This study, which investigates the relationship between vaginal dose and radiation-induced injury, not only addresses a significant gap in the existing research literature but also offers clinicians more targeted guidance for radiotherapy planning. By contributing to the reduction of vaginal injury incidence and meeting practical clinical needs, this study holds substantial innovative value.

Despite the findings of this study on the relationship between vaginal dose and radiation-induced vaginal injury after radiotherapy for cervical cancer, there are still some limitations. First, the sample size was relatively small, with only 66 cervical cancer patients included, which may have affected the stability and reliability of the model. Although the data volume was expanded through data enhancement techniques, the limitation of the original sample size persists, potentially impacting the generalization ability of the model. Second, this study was conducted at a single center, which may introduce selection bias, and the generalizability and applicability of the results require further validation. Additionally, while multiple influencing factors were considered, some potential confounding factors, such as patients’ lifestyle habits and comorbidities, were not included in the analysis, which could impact the occurrence of vaginal radiation injury. Finally, the model validation was primarily based on internal datasets, lacking validation with external independent datasets, which could affect the model’s applicability in different clinical settings. Future studies should aim to expand the sample size, conduct multicenter studies, and incorporate more potentially influential factors to enhance the accuracy and reliability of the model.

In summary, a close relationship exists between PIBS point dose, brachytherapy dose, age, external beam radiation dose, vaginal involvement, and the occurrence of vaginal radiation injury in patients with cervical cancer. Based on these key factors, we have successfully developed a predictive model for vaginal radiation injury. The creation and application of this model not only help to reduce the radiation dose received by the vagina, thereby effectively decreasing the incidence of radiation injury, but also offer significant clinical value in predicting the likelihood of vaginal radiation injury in patients. It provides robust support for the advancement of precise radiotherapy and individualized treatment strategies for cervical cancer.

## Data Availability

The original contributions presented in the study are included in the article/supplementary material, further inquiries can be directed to the corresponding author.
